# Commentary: The immediate pain relief of low-level laser therapy for burning mouth syndrome: a retrospective study of 94 cases

**DOI:** 10.3389/froh.2025.1556733

**Published:** 2025-02-11

**Authors:** Takayuki Suga, Akira Toyofuku

**Affiliations:** Department of Psychosomatic Dentistry, Graduate School of Medical and Dental Sciences, Institute of Science Tokyo, Tokyo, Japan

**Keywords:** burning mouth syndrome, low-level laser therapy, placebo effect, Pharmacotherapy, analgesic efficacy

A Commentary on The immediate pain relief of low-level laser therapy for burning mouth syndrome: a retrospective study of 94 cases By Mu, Li, Lu, Wang and Tao (2024). Front Oral Health. 5. doi: 10.3389/froh.2024.1458329

## 1 Introduction

Dear Editor,

We read with interest the article by Mu et al., entitled “*The immediate pain relief of low-level laser therapy for burning mouth syndrome: a retrospective study of 94 cases*” (1). The authors presented data suggesting a favorable immediate analgesic effect of low-level laser therapy (LLLT) in patients with burning mouth syndrome (BMS). While we commend their effort to explore noninvasive treatment modalities, we would like to raise several points of concern that warrant careful consideration before interpreting their results as definitive evidence of LLLT's efficacy.

## 2 Acknowledgment of LLLT advantages but insufficient placebo control

We fully acknowledge certain advantages of LLLT, including its noninvasive nature, minimal side effects, and ease of application. These features make it an attractive option for patients who are otherwise reluctant to undergo more invasive procedures or long-term pharmacotherapy. However, the authors’ study design without a placebo control group makes it difficult to rule out the possibility of a placebo effect. Although Mu et al. discussed the limitations of their study, their justifications do not exclude the likelihood that at least part of the reported immediate analgesic effect could arise from patient expectation or other nonspecific factors. In this regard, the authors' attempt to equate a short pre-post assessment interval with proper placebo control remains inadequate to definitively confirm a genuine physiological benefit of LLLT.

## 3 Lack of long-term efficacy evaluation

As Mu et al. primarily focused on immediate pain relief, their study design did not address the sustainability of this effect. This is reminiscent of older investigations into BMS, where nerve blocks via infiltrative anesthesia showed temporary benefits but failed to provide conclusive long-term outcomes (2, 3). Despite the possibility that a subset of patients could exhibit clinically meaningful improvements—even if partly influenced by placebo—the lack of long-term data leaves unanswered questions about whether LLLT confers sustained analgesia for BMS. In addition, BMS is recognized as a complex condition often involving psychosomatic and neuropathic components, and short-term pain relief does not necessarily translate into clinically significant long-term remission.

## 4 Standard pharmacotherapy in China and questionable comparisons

It is well recognized—both in our own observation and in many studies—that clonazepam and certain antidepressants are commonly used pharmacological agents for BMS in many settings worldwide (4). In China, the availability of these medications is predominantly restricted to psychiatric practices, creating a notable limitation in drug accessibility for BMS patients. Moreover, the pharmacologic treatments provided to many of their patients (e.g., mecobalamin, basic fibroblast growth factor, oryzanol, etc.) are not consistent with standard BMS medications reported elsewhere. Consequently, claiming that these participants had “standardized” pharmacotherapy prior to receiving LLLT may be an overstatement. The lack of commonly used pharmaceutical therapies (such as clonazepam) in many cases raises the question of whether these patients actually underwent what would be considered standard medical care. If, in fact, the majority of participants did not receive well-recognized treatments, concluding that LLLT succeeds where typical pharmacotherapy fails could be misleading.

## 5 Reports of LLLT non-efficacy and potential overstatement of conclusion

Mu et al. do acknowledge other studies indicating that LLLT may not help all BMS patients and, indeed, may exhibit results comparable to placebo (5). Nonetheless, the overall conclusion of their retrospective study appears overstated in suggesting the near-term pain reduction “proves” LLLT's effectiveness. Given that BMS is highly influenced by psychological factors, the lack of a robust control group, combined with the absence of detailed long-term follow-up, inevitably raises doubts about whether LLLT truly surpasses placebo in clinical practice. The complexity of BMS, involving overlapping neuropathic, psychological, and systemic variables, demands a more cautious interpretation of any single short-term measure of relief.

## 6 Conclusion and recommendations

In light of the concerns outlined above, we urge more rigorous study designs, including prospective randomized controlled trials with adequate placebo controls, standardized drug interventions, and well-defined follow-up periods. We believe such trials would more conclusively determine the extent to which LLLT confers genuine clinical benefit beyond placebo. Additionally, the interplay between psychosomatic variables and peripheral neuropathic mechanisms in BMS underscores the importance of exploring comprehensive, multidisciplinary approaches.

While LLLT may hold promise as part of a broader treatment regimen—particularly in countries where dentists who frequently treat BMS are not legally permitted to prescribe antidepressants or antiepileptic medications and for patients who cannot or prefer not to use pharmacotherapy—the current evidence, as presented by Mu et al., is insufficiently robust to warrant strong claims for routine clinical adoption.

We suggest that future investigations, in accordance with the healthcare context of each country, pay closer attention to recognized pharmacological treatments, employ randomized placebo-controlled methods, and incorporate extended observation intervals to ascertain whether the positive outcomes persist over time. Only then can the field move closer to establishing a more definitive, evidence-based consensus on LLLT for BMS.

Thank you for the opportunity to comment on this intriguing work. We believe that further discussion and additional studies can help refine our understanding of how best to manage this challenging condition.
